# Supplementary material for: Transcriptomic Insights into Mechanisms of Early Seed Maturation in the Garden Pea (Pisum sativum L.)
Source: Cells. 2020 Mar 23;9(3):779. doi: 10.3390/cells9030779 (PMC7140803; doi:10.3390/cells9030779)
Supplement: Supplementary file 1 [file cells-09-00779-s001.zip › Supplementary materials/FileS3.zip!/FileS2/Hormones - Results/Supporting Maps&Profiles/Topology Profiles.pdf]

Number of activated spot modules

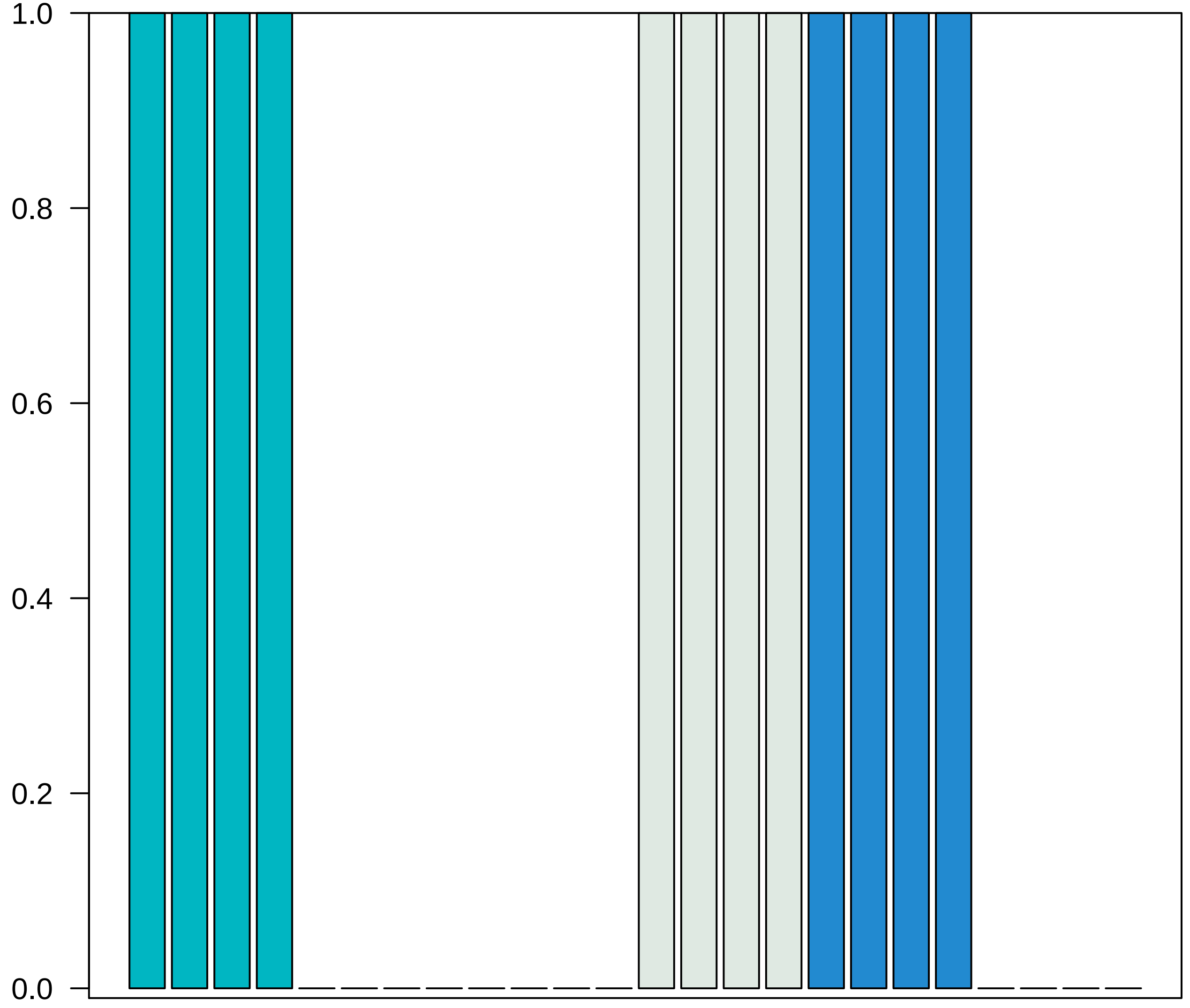

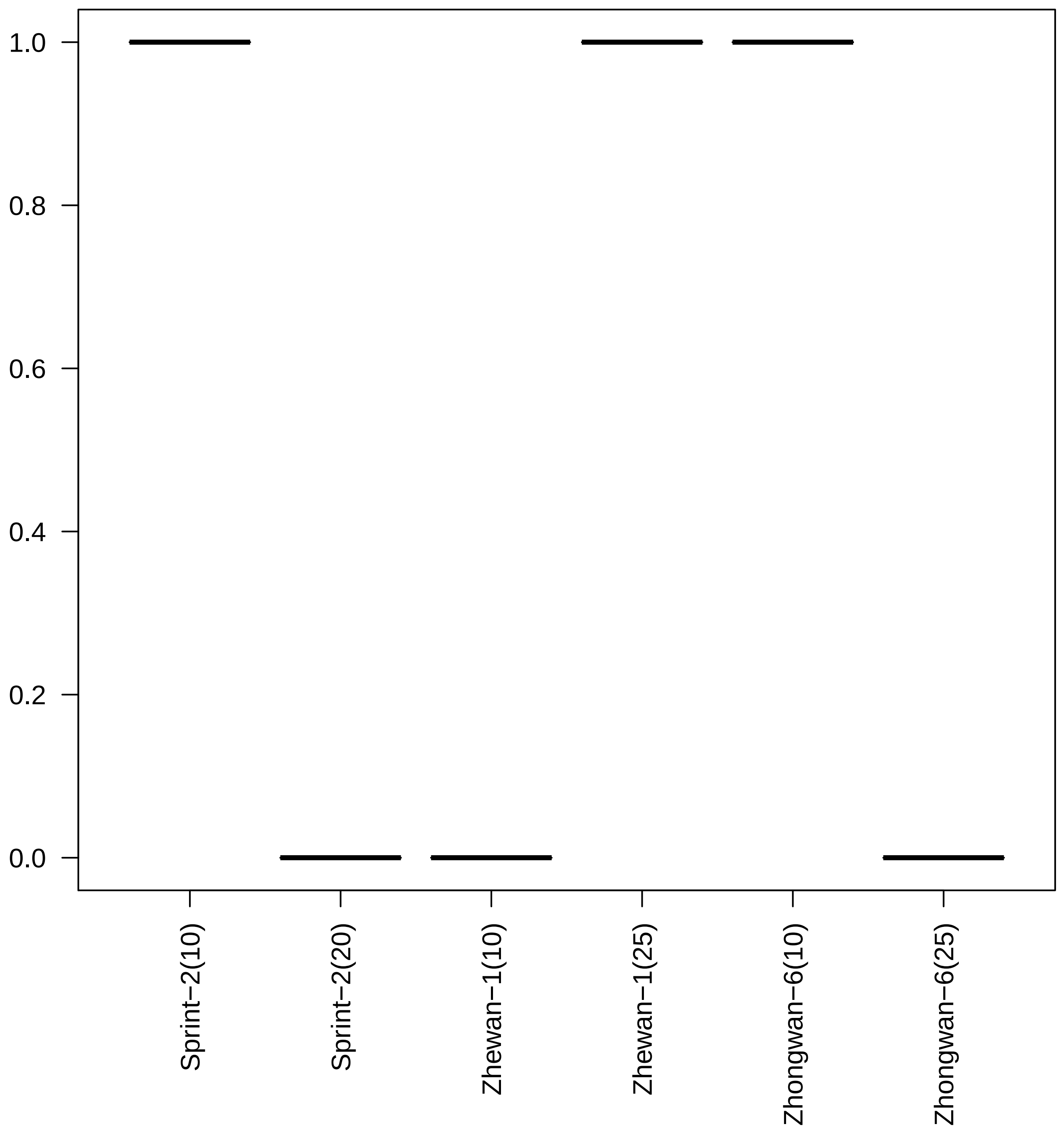

Fraction of samples showing respective number of overexpression spots

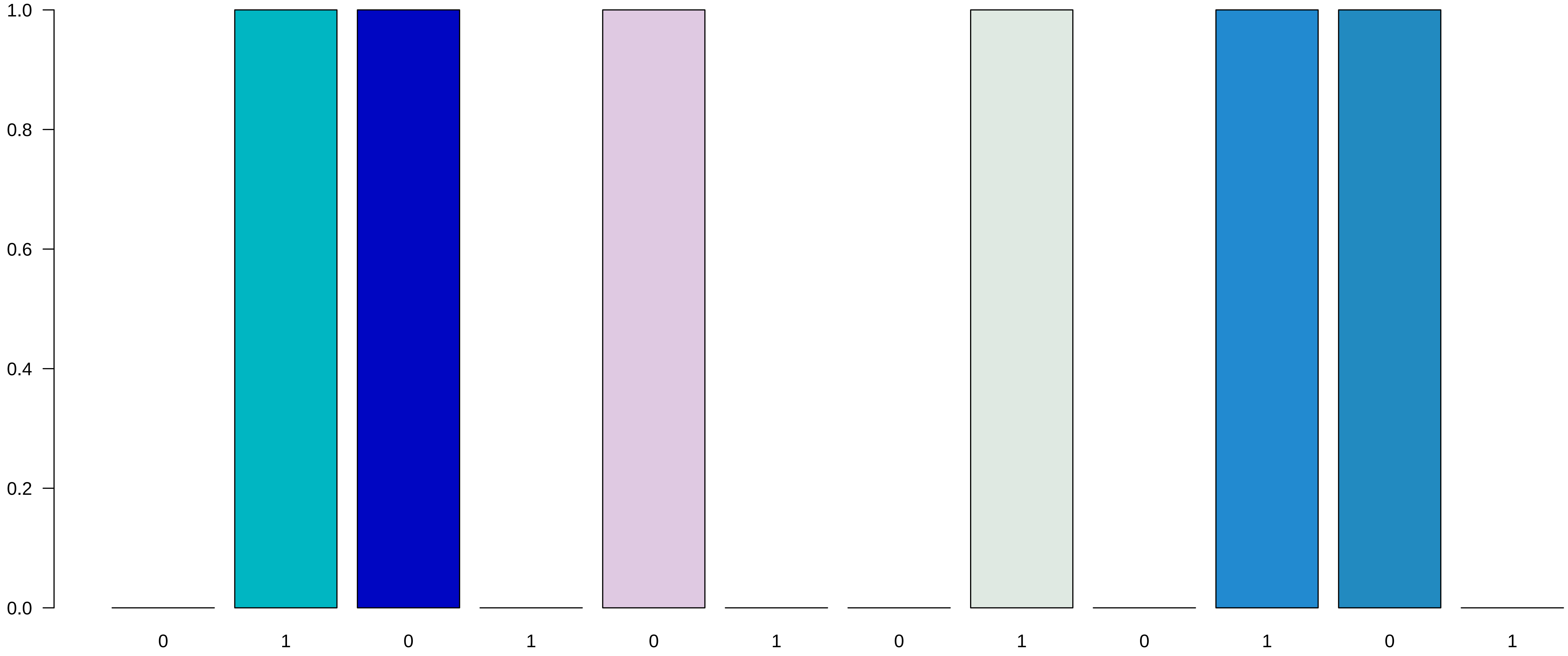

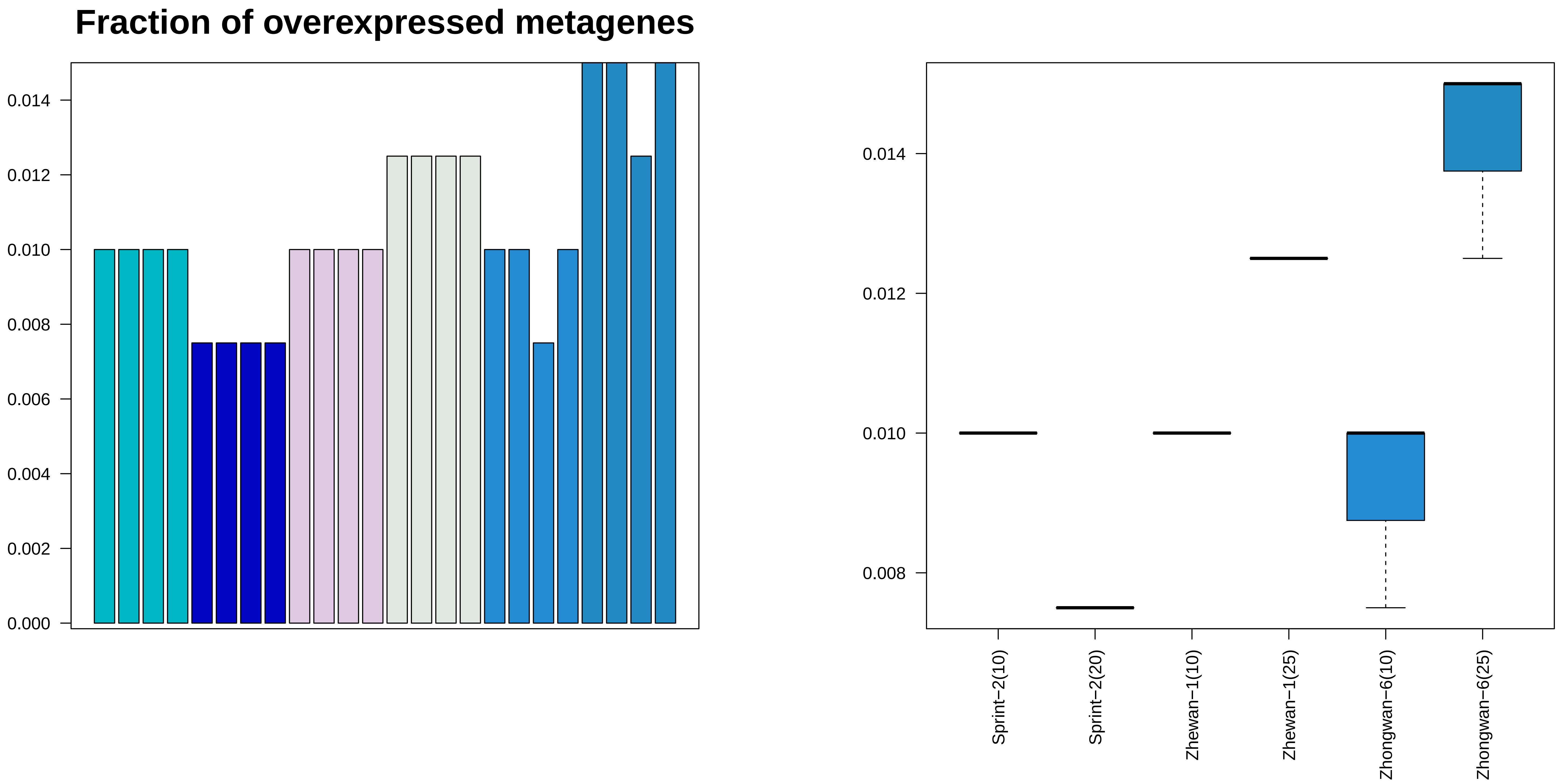
Fraction of overexpressed metagenes
0.000
0.002
0.004
0.006
0.008
0.010
0.012
0.014
0.008
0.010
0.012
0.014
Sprint−2(10)
Sprint−2(20)
Zhewan−1(10)
Zhewan−1(25)
Zhongwan−6(10)
Zhongwan−6(25)

Length of borderline along overexpressed metagenes

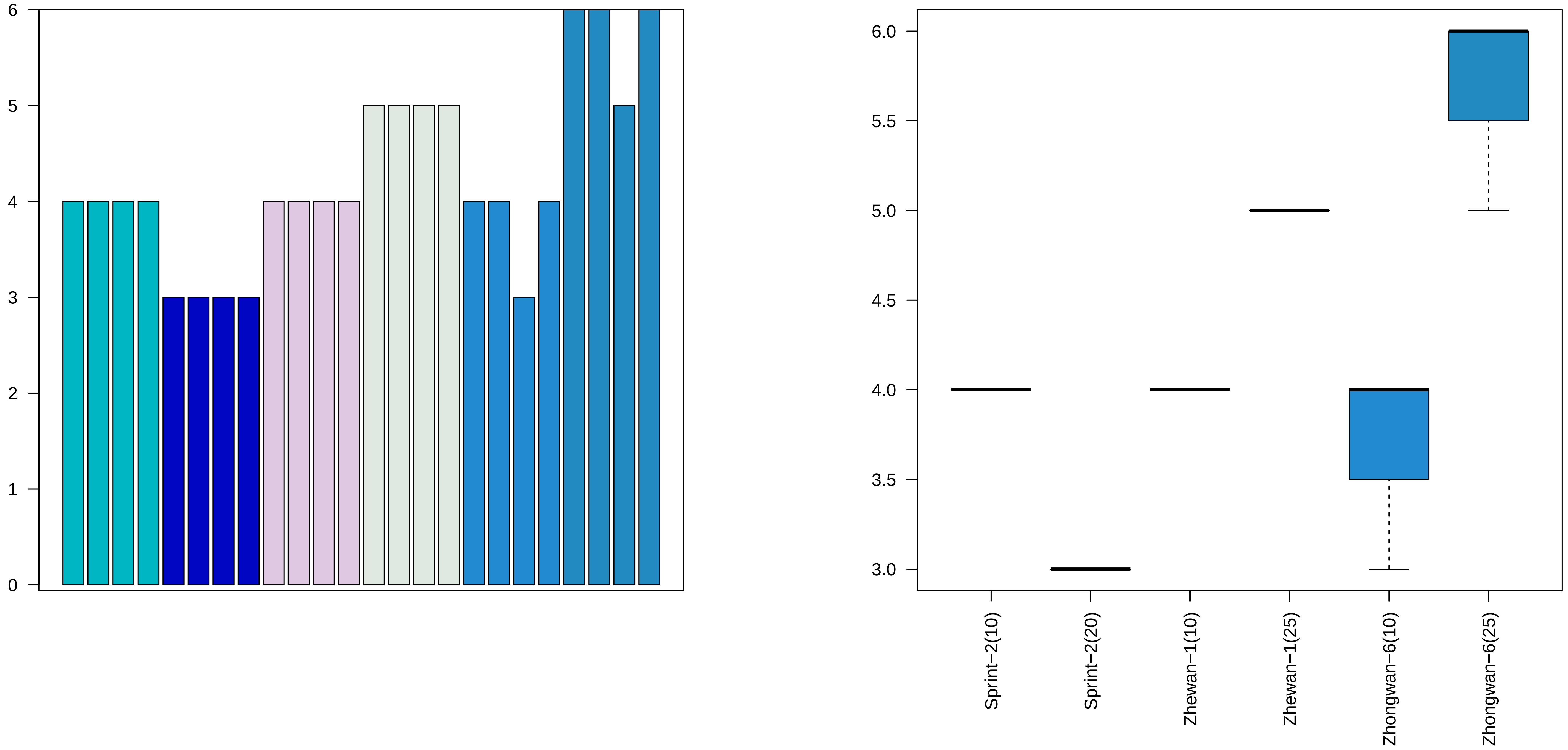

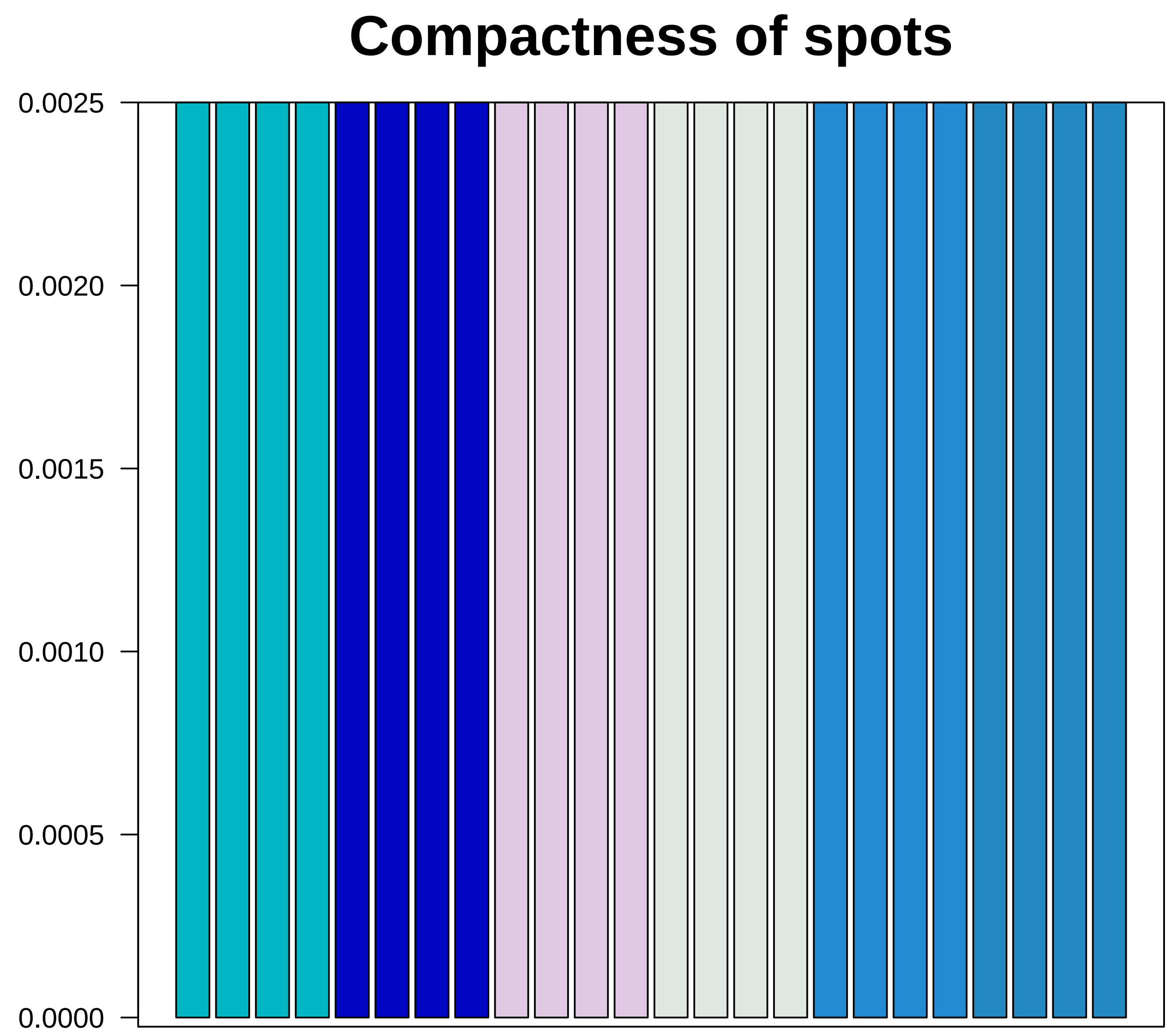

Compactness of spots
0.0025
0.0020
0.0015
0.0010
0.0005
0.0000

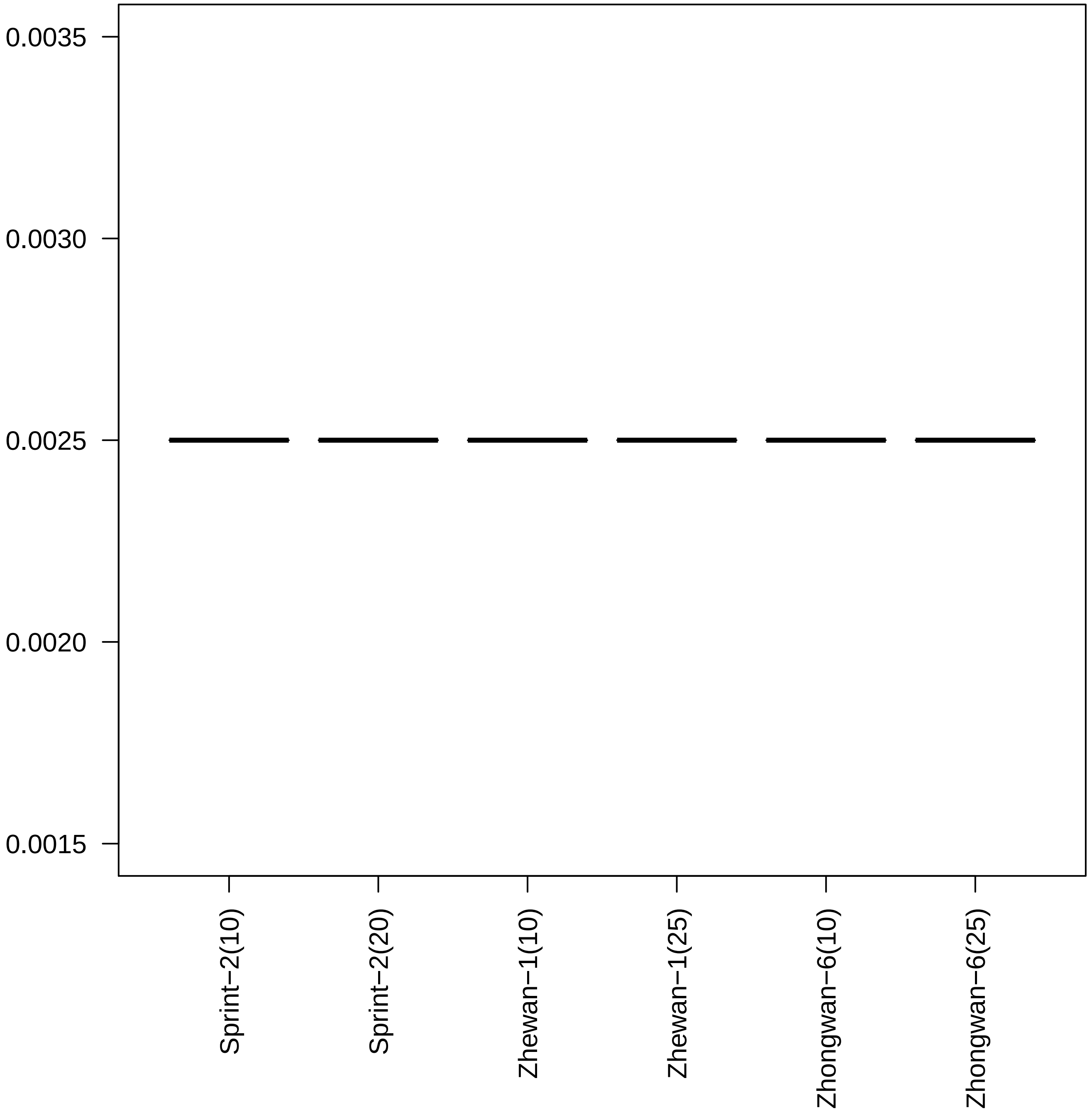

0.0035
0.0030
0.0025
0.0020
0.0015
Sprint−2(10)
Sprint−2(20)
Zhewan−1(10)
Zhewan−1(25)
Zhongwan−6(10)
Zhongwan−6(25)

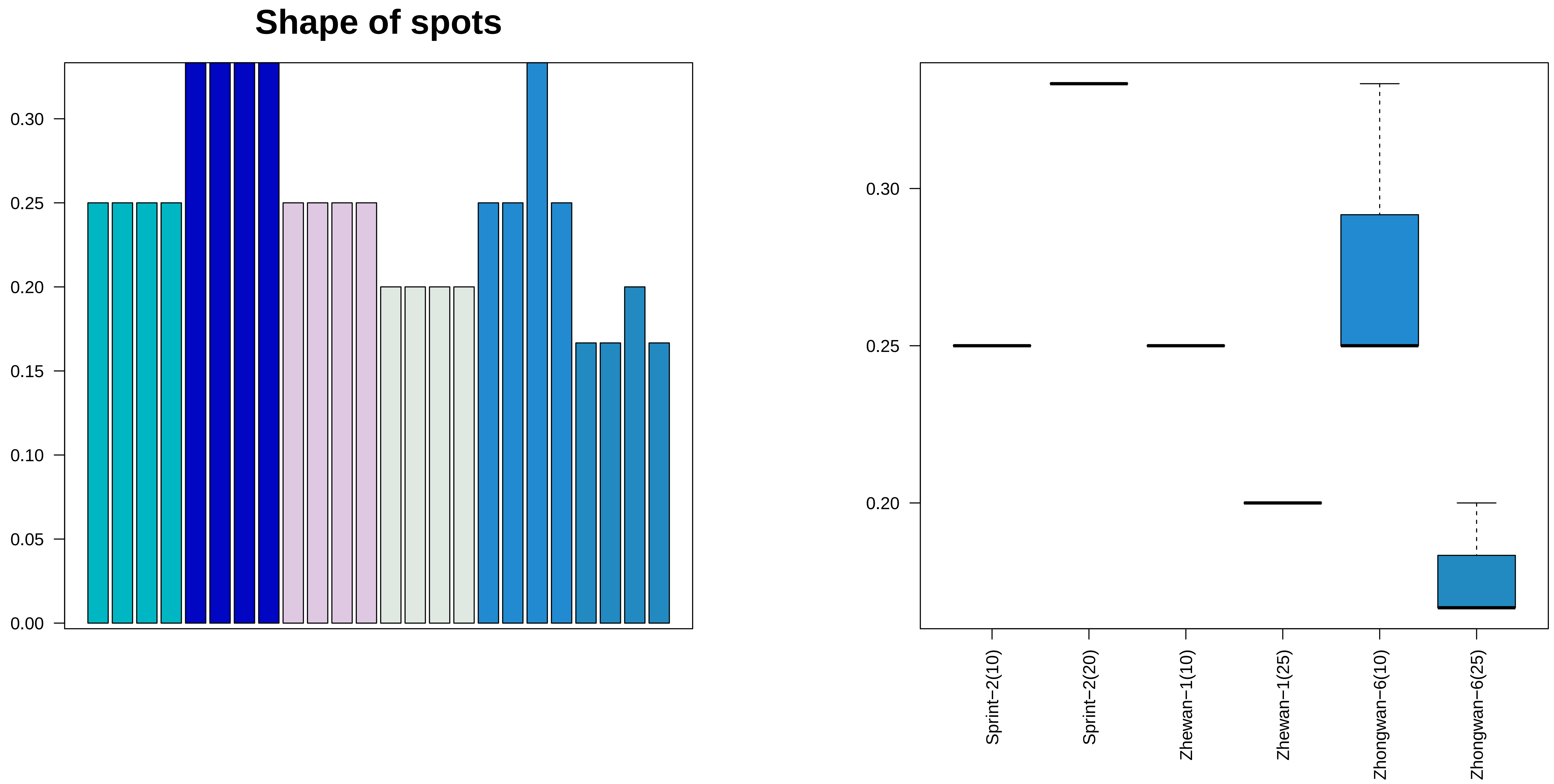
Shape of spots
0.30
0.25
0.20
0.15
0.10
0.05
0.00
0.30
0.25
0.20
Sprint−2(10)
Sprint−2(20)
Zhewan−1(10)
Zhewan−1(25)
Zhongwan−6(10)
Zhongwan−6(25)
